# Characterization of SARS-CoV-2 worldwide transmission based on evolutionary dynamics and specific viral mutations in the spike protein

**DOI:** 10.1186/s40249-021-00895-4

**Published:** 2021-08-21

**Authors:** Jiluo Liu, Xi Chen, Yan Liu, Jiansheng Lin, Jiaying Shen, Hongwei Zhang, Jianhua Yin, Rui Pu, Yibo Ding, Guangwen Cao

**Affiliations:** Department of Epidemiology, Second Military Medical University, 800 Xiangyin Road, Shanghai, 200433 China

**Keywords:** COVID-19, SARS-CoV-2, Evolutionary dynamics, Transmission

## Abstract

**Background:**

The coronavirus disease 2019 (COVID-19) caused by severe acute respiratory syndrome-related coronavirus-2 (SARS-CoV-2) is pandemic. However, the origins and global transmission pattern of SARS-CoV-2 remain largely unknown. We aimed to characterize the origination and transmission of SARS-CoV-2 based on evolutionary dynamics.

**Methods:**

Using the full-length sequences of SARS-CoV-2 with intact geographic, demographic, and temporal information worldwide from the GISAID database during 26 December 2019 and 30 November 2020, we constructed the transmission tree to depict the evolutionary process by the R package “outbreaker”. The affinity of the mutated receptor-binding region of the spike protein to angiotensin-converting enzyme 2 (ACE2) was predicted using mCSM-PPI2 software. Viral infectivity and antigenicity were tested in ACE2-transfected HEK293T cells by pseudovirus transfection and neutralizing antibody test.

**Results:**

From 26 December 2019 to 8 March 2020, early stage of the COVID-19 pandemic, SARS-CoV-2 strains identified worldwide were mainly composed of three clusters: the Europe-based cluster including two USA-based sub-clusters; the Asia-based cluster including isolates in China, Japan, the USA, Singapore, Australia, Malaysia, and Italy; and the USA-based cluster. The SARS-CoV-2 strains identified in the USA formed four independent clades while those identified in China formed one clade. After 8 March 2020, the clusters of SARS-CoV-2 strains tended to be independent and became “pure” in each of the major countries. Twenty-two of 60 mutations in the receptor-binding domain of the spike protein were predicted to increase the binding affinity of SARS-CoV-2 to ACE2. Of all predicted mutants, the number of E484K was the largest one with 86 585 sequences, followed by S477N with 55 442 sequences worldwide. In more than ten countries, the frequencies of the isolates with E484K and S477N increased significantly. V367F and N354D mutations increased the infectivity of SARS-CoV-2 pseudoviruses (*P* < 0.001). SARS-CoV-2 with V367F was more sensitive to the S1-targeting neutralizing antibody than the wild-type counterpart (*P* < 0.001).

**Conclusions:**

SARS-CoV-2 strains might have originated in several countries simultaneously under certain evolutionary pressure. Travel restrictions might cause location-specific SARS-CoV-2 clustering. The SARS-CoV-2 evolution appears to facilitate its transmission via altering the affinity to ACE2 or immune evasion.

**Graphic Abstract:**

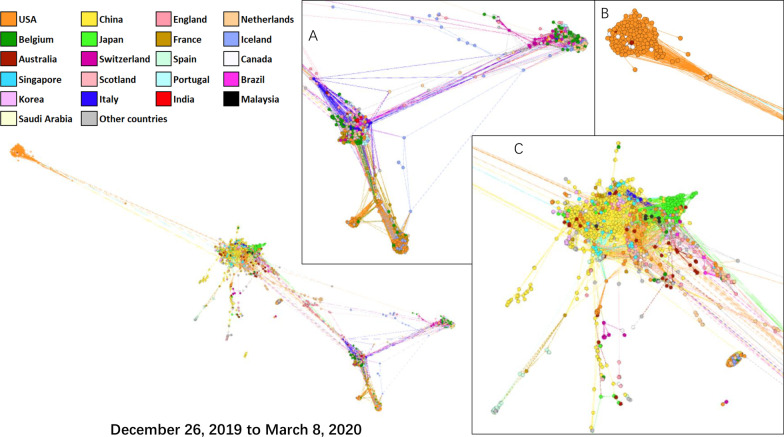

**Supplementary Information:**

The online version contains supplementary material available at 10.1186/s40249-021-00895-4.

## Background

Coronaviruses (CoVs), a genus within the Coronaviridae family, consists of four genera—α-CoV, β-CoV, γ-CoV, and δ-CoV—according to their phylogenetic relationships and genomic structures. Before 2002, only four kinds of coronaviruses (HCoV-NL63, HCoV-229E, HCoV-OC43, and HKU1) were known to infect humans and cause mild upper respiratory infection in 10–30% of adults, as well as, occasionally, severe pneumonia in the elderly, infants, and immunodeficient persons [1]. During 2002–2003, severe acute respiratory syndrome CoV (SARS-CoV) infected 8098 persons globally, with a case fatality rate of 9.6%; during 2012–2015, Middle East respiratory syndrome CoV (MERS-CoV) infected 2494 persons globally, with a case fatality rate of 34.4% [1, 2]. As causative agents of novel natural focus diseases, SARS-CoV and MERS-CoV belong to β-CoV [2, 3]. A novel coronavirus disease, the corona virus disease 2019 (COVID-19) caused by severe acute respiratory syndrome-related coronavirus-2 (SARS-CoV-2), was first identified on 26 December 2019 [4]. SARS-CoV-2 is a new member of β-CoV subfamily, with an RNA genome of 29 kb. Although the similarity in genomic sequences between SARS-CoV-2 and SARS-CoV is only 79.6%, the two β-CoVs share the same receptor—angiotensin converting enzyme II (ACE2) [4]. SARS-CoV-2 was suggested to originate from the bat host and Malayan pangolin is suspected to be an intermediate host of SARS-CoV-2 [4, 5]. However, no solid evidence confirms the natural hosts and intermediate host of SARS-CoV-2.

COVID-19 was first diagnosed in the USA on January 19, 2020 [6]. Since then, the number of COVID-19 cases has continually increased globally and become a pandemic. As an RNA virus, SARS-CoV-2 often mutates. Soon after the outbreak, SARS-CoV-2 mutations including D614G appeared [7]. SARS-CoV-2 infects humans via binding to its receptor ACE2, a key step in cell entry. The high-affinity binding of the spike (S) protein to human ACE2 is an essential prerequisite for rapid transmission of SARS-CoV-2 in humans. The strains with mutations at the ACE2 binding site including Alpha (B.1.1.7), Beta (B.1.351), Gamma (P.1), and Delta (B.1.617.2) increase viral infectivity and immune evasion, thus becoming regional adaptive strains [8, 9]. The affinity of the S protein binding to human ACE2 reflects the direction of SARS-CoV-2 evolution in humans. It is important to identify the specific mutations worldwide, especially the mutations in the S protein, and their changing affinity to ACE2. However, origination, evolution, and transmission patterns of SARS-CoV-2 remain largely unknown.

Whole genome sequencing, phylogenetic analysis, and transmission reconstruction of pathogens are important tools and promising approaches for understanding the spread of infectious diseases in near real time, allowing to pinpoint outbreak origins and to resolve transmission patterns at multiple geographic scales [10–13]. Here, we conducted bioinformatics analysis to speculate possible recombination, origins and transmission processes of SARS-CoV-2 and evaluate the influence of mutations in the S protein on the transmission of SARS-CoV-2. Then, cell experiments were performed to evaluate the effects of specific viral mutations on the infectivity and immunoreaction of neutralizing antibody against SARS-CoV-2. This study helps elucidate the evolution of SARS-CoV-2 and develop suitable prophylactic options to fight against COVID-19.

## Methods

### Retrieval of SARS-CoV-2 full-length sequences worldwide

All full-length sequences or segments of human SARS-CoV-2 updated to 30 November 2020 were retrieved from the GISAID database (https://www.gisaid.org/) [14]. To reconstruct the transmission network of SARS-CoV-2, we included the full-length sequences of SARS-CoV-2 according to the following criteria: (i) the sequences with information of geographic locations where the viruses were identified; (ii) those with the dates of collection and with available information of patients; (iii) the genome length of > 29 000 bp; (iv) undefined bases < 1%; and (v) no insertion or deletion unless verified by submitters. In total, 8795 of the 230 103 sequences met the criteria and were all included in the evolutionary analysis.

The number of confirmed cases of COVID-19 surpassed 100 000 globally and the World Health Organization (WHO) declared COVID-19 as a pandemic on early March 2020 (https://www.who.int/news/item/29-06-2020-covidtimeline). Therefore, we defined the period from 26 December 2019 to 8 March 2020 as the early stage of the pandemic. Of the 8795 full-length SARS-CoV-2 strains included in evolutionary analysis, 1861 were harvested at this stage.

### Recombination analysis

Highly similar full-length of CoV sequences from animal source including bats, palm civets, mice, dogs, pigs, birds, and pangolins up to February 2020 to the SARS-CoV-2 (EPI_ISL_406798) isolated in Wuhan, China were automatically searched on BLAST (http://www.ncbi.nlm.nih.gov/BLAST/Blast.cgi). Three full-length CoVs from pangolins were also derived from GISAID database (https://www.gisaid.org/) (access date: 29 February, 2020) [14]. In total, 51 CoVs from animal hosts were retrieved for recombination analysis (Additional file 1: Table S1). Potential recombination events were determined using RDP, GENECONV, BootScan, maximum chi-square, Chimera, SISCAN, and Phylpro methods integrated in the Recombination Detection Program v4.99 (RDP 4) (http://web.cbio.uct.ac.za/~darren/rdp.html) [15]. Potential recombination events with Bonferroni *P*-value < 0.01 were identified and visualized by Simplot v3.5.1 (The Johns Hopkins School of Medicine, Baltimore, MD, USA) [16].

### Quantitative monitoring of SARS-CoV-2 strains

For monitoring the quantity change of mutant strains, specific sequences or segments were counted through the online tool offered by GISAID, allowing to count the exact number of specific mutants in certain locations and periods (https://www.gisaid.org/) [14]. The quantitation changes of local SARS-CoV-2 mutant strains were monitored as previously reported [7]. Briefly, the onset time of the local epidemic of mutant strains referred to the date when the cumulative number of specific sequences reached 15. Relevant mutants were analyzed only when the numbers of certain strains reached 100 locally by the deadline (30 May, 2021). The comparison between the proportions of mutant strains before and after the onset time was made by the two-sided Fisher’s exact test.

### Reconstruction and visualization of transmission tree

The R package “outbreaker”, a statistical method exploiting viral genetic sequences and collection dates, was applied to reconstruct the transmission tree [17]. The sequences in the evolutionary dynamics analysis included 1861 strains from 26 December, 2019 to 8 March, 2020; 1432 strains from 9 to 31 March, 2020; 1476 strains from 1 to 30 April, 2020; 1591 strains from 1 May to 30 June, 2020; 1447 strains from 1 July to 31 August, 2020; and 988 strains from 1 September to 30 November, 2020. Among those 8795 sequences, 2000 were randomly selected to depict the evolution network from 26 December 2019 to 30 November, 2020, according to stratified randomization by quantity in each month and a table of random numbers. By combining genomic sequences and collection dates of SARS-CoV-2, network analysis of the viral evolutionary process was performed. Gephi 0.9.2 software (Gephi Consortium 2010) was applied for network visualization [18]. The Force Atlas and Fruchterman Reingold models were applied to align isolates in Gephi. In the network, COVID-19 patients were set as nodes, whose colors represented locations. The distance between clades represented evolutionary distances. Colors of lines between clades represented the direction of evolution. Lines inherited colors from parental clades.

### Predicting amino acid mutations and their effect

The 1861 strains from early stage of the SARS-CoV-2 pandemic were selected to analyze amino acid mutations and predict the change of the affinity to ACE2 compared to the reference sequence. Affinity changes were also predicted among strains collected after the early stage, using 2000 randomly selected sequences collected from 9 March, 2020 to 30 November, 2020. The selection of 2000 sequences was finished according to stratified randomization by quantity in each month and a table of random numbers. To summarize amino acid mutations, Glimmer v3.02 was applied to analyze the open reading frames (ORFs) of the S protein of SARS-CoV-2 from nucleotide sequences [19]. ORFs were extracted and translated into amino acids by Bioperl [20]. Multiple sequence alignment was performed for the S proteins by MUSCLE 3.8.31 [21]. Taking the sequence of EPI_ISL_406798 as a reference, we extracted acid amino mutations of the included sequences. The dimer structure of the S protein and ACE2 [in the format of protein data bank (pdb)] was downloaded from the National Microbiology Data Center (accession: NMDCS0000001). Mutations in the receptor-binding domain (RBD) were taken as the input of mCSM-PPI2 (http://biosig.unimelb.edu.au/mcsm_ppi2/) to predict the change of free binding energy, as previously described [22].

### Cell lines

HEK293T cells were purchased from the American Type Culture Collection (ATCC, VA, USA). HEK293T cells stably overexpressing the receptor for the SARS-CoV-2 S protein, ACE2, (HEK293T-ACE2) were purchased from Yeasen Biotech (Shanghai, China, 41107ES03). Both HEK293T and HEK293T-ACE2 cells were cultured in high glucose Dulbecco's modified Eagle's medium (DMEM, Hyclone, MA, USA) with 10% fetal bovine serum (FBS, Rockville, MD, USA), 100 U/ml penicillin, and 100 µg/ml streptomycin in 5% CO_2_ at 37 °C.

### Production and titration of SARS-CoV-2 S pseudoviruses

SARS-CoV-2 S pseudotyped virus with wild-type spike sequence and the N354D and V367F mutants were purchased from Yeasen (wild-type Cat. Nos. 11906ES50, V367F Cat. Nos.18101ES50, and N354D Cat. Nos.18103ES70). The final titer of each of these three pseudoviruses was 1 × 10^7^ TU/ml. In brief, a lentivirus-based vesicular stomatitis virus (VSV) system was applied to generate the SARS-CoV-2 S pseudovirus. The full-length SARS-CoV-2 spike gene (Wuhan-Hu-1 isolate, GenBank: NC_045512.2) was codon-optimized, synthesized, and cloned into the GPLVX vector carrying the luciferase gene and the 2sGreen gene (GPLVX-CMV-2sGreen-T2A-Luc). V367F and N354D mutants were generated by site-directed mutagenesis. The recombinant SARS-CoV-2 S plasmids containing wild type spike or the S mutants (SARS-CoV-2 S V367F and SARS-CoV-2 S N354D) were verified by DNA sequencing (Additional file 2: Fig. S1). HEK293T cells (6 × 10^6^ cells per 10-cm plate) were co-transfected with recombinant SARS-CoV-2S plasmids (wild type, V367 mutant, or N354D mutant) and the packaging vectors (pLP1, pLP2, and pLP/VSVG, Invitrogen) using liposomal transfection reagent (Yeasen, No. 40802ES). Ten hours after the transfection, enhancing buffer (Yeasen, 40804ES) was added according to manufacturer’s instructions. Eighteen hours after the transfection, the culture medium was replaced with fresh one. Forty-eight hours later, the supernatant containing SARS-CoV-2 S pseudovirus was harvested and filtered through a 0.45-μm filter.

Spike-pseudotyped lentiviral particles were quantitated using HEK293T-ACE2 cells. The cells were seeded in 96-well plates at 1 × 10^4^/well. Then, a serial tenfold diluted pseudovirus was added to the cultures to infect cells. Six hours later, the supernatants were removed and replaced with fresh culture medium. Forty-eight hours later, the pseudovirus titer was measured by counting the cells expressing green fluorescent protein under a fluorescence microscope. The measured titer was expressed as transduction units per milliliter (TU/ml).

### Pseudovirus infectivity assay and neutralization assay

Pseudovirus infectivity directly corresponded to the relative luminescent units (RLUs) produced by the luciferase gene incorporated into the pseudovirus genome. HEK293T-ACE2 cells were seeded in 96-well plates at 1.5 × 10^4^/well, and 3700 TU pseudoviruses (wild type, V367F mutant, or N354D mutant) were added to the culture medium. Six hours after the infection, the culture medium was replaced with fresh DMEM. Forty-eight hours after the infection, luciferase activity was measured using a luciferase assay kit (Yeasen, No. 11401ES60) according to the manufacturer’s instructions.

Neutralization was measured as reduction in luciferase gene expression, as previously described [23]. In brief, pseudoviruses (0.37 µl, 1 × 10^7^ TU/ml) were incubated with serially diluted SARS-CoV-2 spike neutralizing antibody (SinoBiological, No. 40592-MM57) (30 μg/ml, 10 μg/ml, 3.33 μg/ml, 1.11 μg/ml, 370.37 ng/ml, 123.46 ng/ml, 41.15 ng/ml, 13.72 ng/ml, and 4.57 ng/ml) for 1 h at room temperature. Then, the pseodoviruses were added to wells seeded with HEK293-ACE2 cells at 1.5 × 10^4^ cells/well. Six hours after the infection, the culture medium was replaced with fresh DMEM. Luciferase assays were performed 48 h post-infection. The 50% inhibitory effect (IC_50_) was defined as the concentration of SARS-CoV-2 spike neutralizing antibody at which the RLU was reduced by 50% compared with the control (without neutralizing antibody).

### Statistical analysis

GraphPad Prism 6.0 software and Statistical Package for Social Sciences (SPSS) version 21.0 (IBM Corp., Armonk, NY) were applied to perform all statistical analyses. The proportions of mutant strains before and after the onset time in different countries were compared by the two-sided Fisher’s exact test. The data in the in vitro experiments are presented as mean value and standard deviations (*SD*s) and student *t*-test was performed for two-group comparisons. Differences with *P*-values < 0.05 were deemed statistically significant.

## Results

### Potential recombination with CoVs from natural reservoirs

**Fig. 1:**
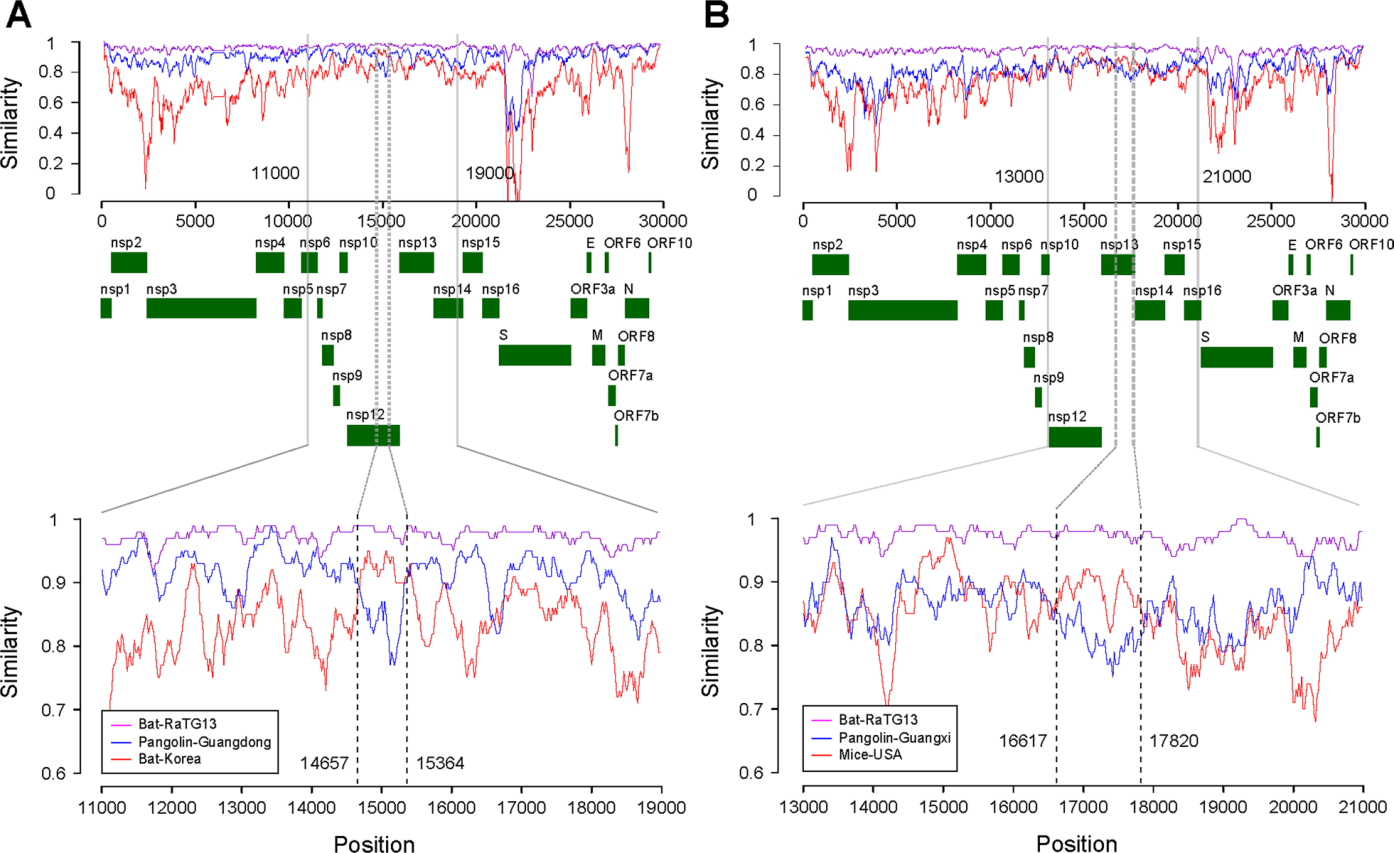
Potential recombination events in the full-length SARS-CoV-2 genome. The purple curves represented the sequence of RaTG-13. Two possible recombination events were detected: **A** between a CoV from a bat from abandoned mine in South Korea in 2016 (KY938558) and a CoV from a pangolin collected in Guangdong in 2019 (EPI_ISL_410721) at nt.14657–nt.15364 (Bonferroni *P*-value < 0.01) and **B** between a SARS-like CoV from experimental BALB/c mice in the USA in 2008 (FJ882945) and a CoV from a pangolin collected in Guangxi in 2017 (EPI_ISL_410539) at nt.16617–nt.17820 (Bonferroni *P*-value < 0.01)

The sequences of included CoVs of animal resources were presented in Additional file 1: Table S1. The CoV shared the best similarity to SARS-CoV-2 in the full-length genome was Bat-RaTG13 [4]. Even though, nucleotide variations were equally distributed in the full-length genome of CoVs (Fig. 1). Although two possible recombination events were detected, the recombination might not be real due to the relatively low sequence similarities and geographic separation. These data indicate that the SARS-CoV-2 is a naturally evolved CoV.

### Evolutionary analysis of global transmission network of SARS-CoV-2

**Fig. 2:**
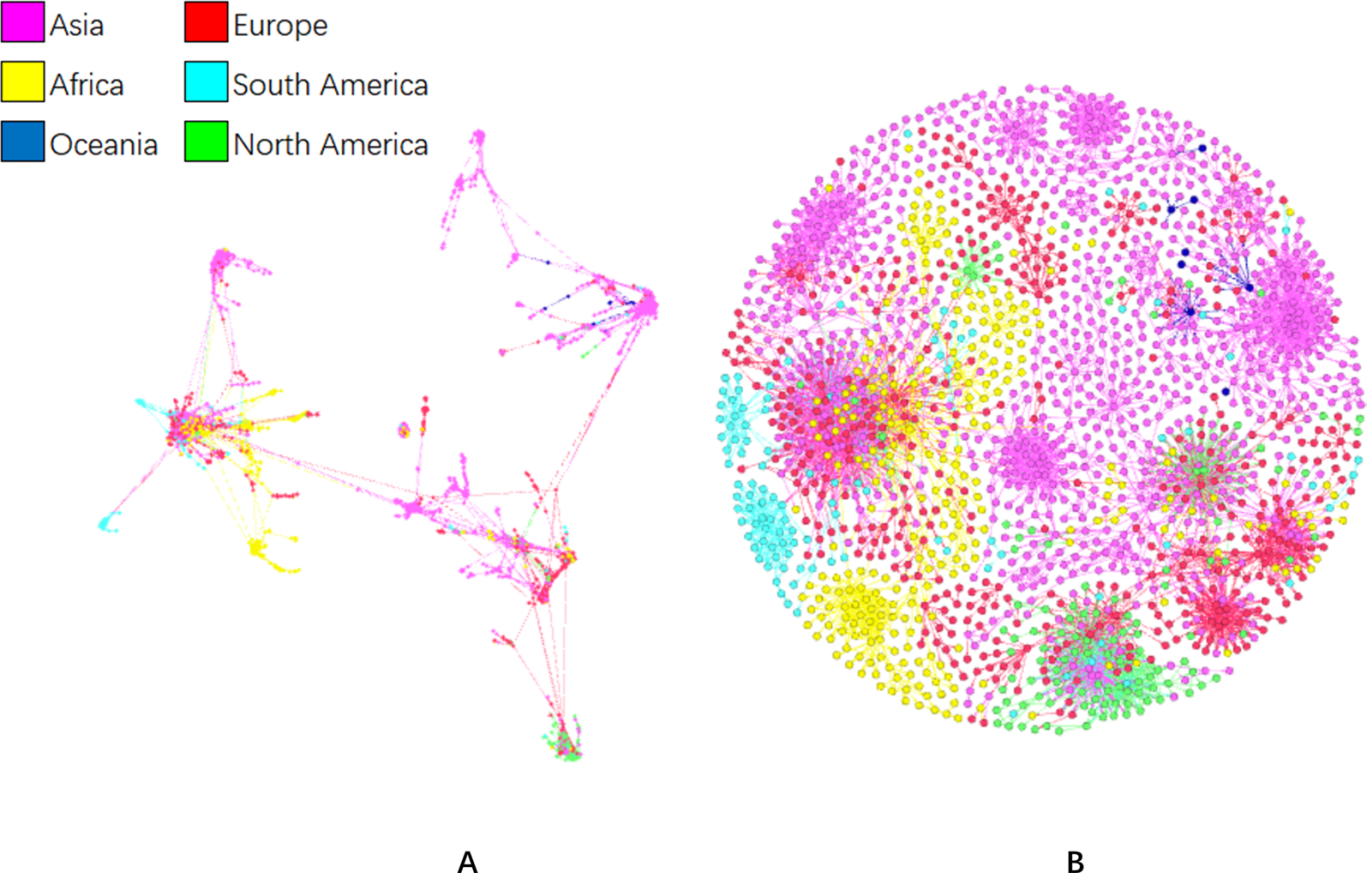
Graphic representation of the transmission network generated in Gephi. Two thousand full-length SARS-CoV-2 sequences were randomly selected from the 8795 sequences collected from 26 December, 2019 to 30 November, 2020. In the network, each node represented an isolate of SARS-CoV-2. Each color represented a continent. Lines inherit colors from their origin clades. Distances between clades represented evolutionary distance. Isolates were aligned by: **A** the model of Force Atlas in Gephi; and **B** the model of Fruchterman Reingold

We first evaluated the transmission network using 2000 representative SARS-CoV-2 strains randomly collected from the 8795 sequences collected during 26 December, 2019 and 30 November, 2020. It was found that the 2000 SARS-CoV-2 strains were clustered into three groups (Fig. 2A). SARS-CoV-2 strains that clustered together were mainly identified on the same continent (Fig. 2B).

**Fig. 3:**
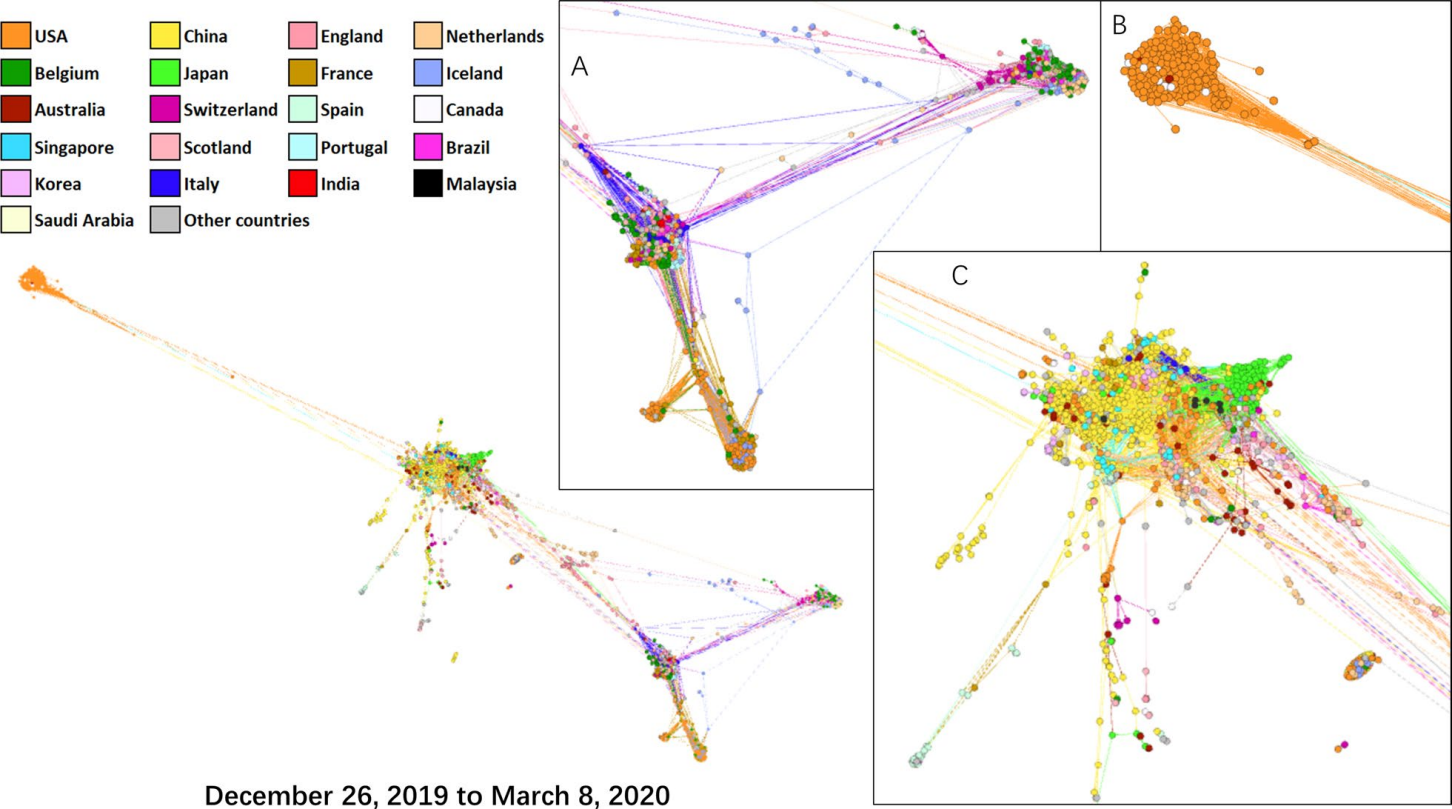
Network graphic of SARS-CoV-2 isolates worldwide during 26 December, 2019 and 8 March, 2020. Isolates were aligned by the Force Atlas model in Gephi. In the network, each node represented an isolate of SARS-CoV-2. Each color represented a country. Lines inherit colors from their origin clades. Distances between clades represented evolutionary distance

We then evaluated the transmission network using all the full-length SARS-CoV-2 strains collected worldwide at different stages during 2019–2020. At the early stage of the COVID-19 outbreak (26 December, 2019–8 March 2020), SARS-CoV-2 strains across the world were mainly composed of three clusters (Fig. 3). Viruses identified in Italy and England constituted the core of Cluster A. The strains in Cluster A were further divided into four clades: mainly identified in the USA, Italy, the Netherlands, Belgium, England, Scotland, and Brazil. Viral strains in Cluster B were mainly identified in the USA, with only several viruses identified in Canada and Australia. Cluster C contained the strains identified in China, Japan, the USA, Singapore, Australia, Malaysia, and Italy. Strains of Clusters A and B were phylogenetically linked to Cluster C; however, the evolutionary distance between Clusters B and C was longer than that between Clusters A and C. Thus, SARS-CoV-2 strains identified in the USA had at least four independent clades, in which Cluster B was the major one. The colors of the links between Clusters B and C were mostly the same as the main color of Cluster B, indicating that the different clusters of SARS-CoV-2 identified in the USA were cross-linked.

The strains collected from 9 to 31 March, 2020 were divided into three major clusters (Additional file 3: Fig. S2). Unlike the situation in the early stage of the COVID-19 pandemic, the clusters of SARS-CoV-2 strains were mostly independent of each other, especially for the evolutionary relationship between Clusters A and B or among Clusters C, D, and E. The situation was also observed in the subsequent months. Clusters A and C contained SARS-CoV-2 strains identified in the USA, Israel, France, and Singapore, forming the first major group. The strains in Cluster C were identified in Vietnam, China, Italy, Brazil, France, and Spain. The strains in Clusters B and E were independent, having no evolutionary relationship with other groups. The strains in Cluster B were identified in Russia, Italy, Brazil, and Japan.

The evolutionary pattern of global SARS-CoV-2 showed more obvious characteristics of clustering after April 2020. The color of the isolates in each clade was becoming pure, indicating that the SARS-CoV-2 variants in a given country tend to cluster together (Additional file 4: Fig. S3).

From 1 May to 31 August, 2020, the pandemic mitigated. SARS-CoV-2 strains were more identified in India (Clusters B and C) and Singapore (Clusters A and D) (Additional file 5: Fig. S4). Cluster C also contained the strains identified in Saudi Arabia, South Africa, and the USA, while Cluster B also contained strains identified in Brazil and Italy.

The strains identified in India and South Africa formed several clusters in June and August 2020 (Additional file 6: Fig. S5). The strains identified in South Africa shaped the core of Clusters A and D. Strains identified in South Africa and India formed Clusters B and C, two clusters with a weak link.

**Fig. 4:**
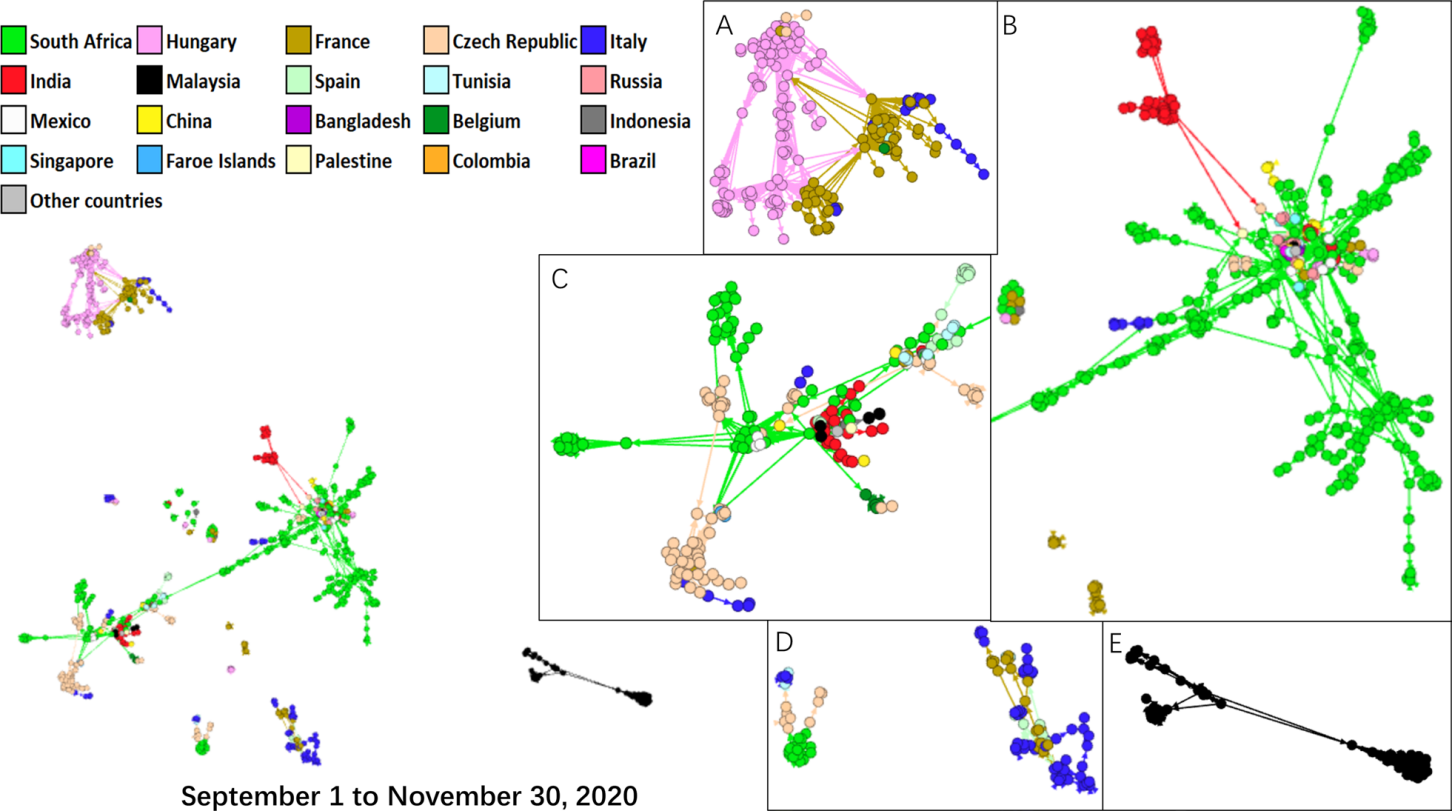
Network graphic of SARS-CoV-2 isolates worldwide during 1 September and 30 November, 2020. Isolates were aligned by the Force Atlas model in Gephi. In the network, each node represented an isolate of SARS-CoV-2. Each color represented a country. Lines inherit colors from their origin clades. Distances between clades represented evolutionary distance

Figure 4 shows the evolutionary relationship of the SARS-CoV-2 strains globally from 1 September to 30 November, 2020. Cluster A contained the SARS-CoV-2 strains identified in Hungary, France, and Italy. It had no links with other clusters. The SARS-CoV-2 strains identified in South Africa were the main strains in Clusters B and C. Cluster C also contained strains identified in Czech Republic, India, Tunisia, Italy, and Belgium. The strains identified in India, Russia, Italy, Mexico, France, and China were also included in Cluster C. The strains identified in Italy and France (Cluster D) and those in Malaysia (Cluster E) formed independent clades.

### The effect of the S protein mutations on the binding to ACE2

We identified possible mutations in the S protein of SARS-CoV-2 and then estimated the effect of these mutations on the affinity of the S protein binding to ACE2. The S genes from all available SARS-CoV-2 sequences were identified. Among sequences reported at the early stage, we extracted 38 amino acid mutations located within the RBD region of the S protein. Based on sequences reported after 8 March, 2020, 26 amino acid mutations (4 were previously predicted) were extracted. We predicted that the binding free energy of the S proteins in 12 of the 38 mutations at early stage and 12 of the 26 mutations after early stage decreased (affinity increased) (Table 1). This result indicates that some mutations increase the binding affinity of the S protein to ACE2, thus facilitating the transmission of SARS-CoV-2 in humans.

**Table 1:** Change in binding free energy related to mutations in receptor-binding domain of the spike protein

Wild type	Position	Mutant type	Mutants	Distance to interface	ΔΔG_wild-mutation_ (kcal/mol)	Affinity
From 26 December, 2019 to 8 March, 2020						
ARG	509	LYS	R509K	15.117	-0.78	Decreasing
PHE	338	LEU	F338L	25.998	-0.776	Decreasing
TYR	508	HIS	Y508H	8.916	-0.595	Decreasing
GLU	516	GLN	E516Q	32.457	-0.556	Decreasing
HIS	519	PRO	H519P	40.899	-0.534	Decreasing
HIS	519	GLN	H519Q	40.899	-0.502	Decreasing
GLY	476	SER	G476S	3.806	-0.433	Decreasing
ASN	439	LYS	N439K	7.234	-0.331	Decreasing
ASP	467	VAL	D467V	14.986	-0.291	Decreasing
VAL	510	LEU	V510L	16.548	-0.27	Decreasing
GLN	409	GLU	Q409E	9.46	-0.253	Decreasing
LYS	378	ARG	K378R	19.35	-0.245	Decreasing
ARG	408	ILE	R408I	10.586	-0.199	Decreasing
ASP	405	VAL	D405V	7.48	-0.196	Decreasing
ILE	468	THR	I468T	14.973	-0.177	Decreasing
ILE	472	VAL	I472V	8.123	-0.177	Decreasing
SER	477	GLY	S477G	4.97	-0.172	Decreasing
GLY	446	VAL	G446V	3.279	-0.158	Decreasing
GLN	414	ALA	Q414A	15.173	-0.118	Decreasing
LYS	458	ASN	K458N	9.745	-0.089	Decreasing
VAL	483	ALA	V483A	9.719	-0.053	Decreasing
SER	438	PHE	S438F	11.54	-0.039	Decreasing
VAL	483	ILE	V483I	9.719	-0.026	Decreasing
ALA	372	SER	A372S	15.674	-0.023	Decreasing
ASP	364	TYR	D364Y	28.736	-0.021	Decreasing
ALA	475	VAL	A475V	2.813	-0.001	Decreasing
ALA	522	VAL	A522V	41.819	0.024	Increasing
PRO	491	ARG	P491R	6.013	0.033	Increasing
LYS	458	ARG	K458R	9.745	0.042	Increasing
VAL	341	ILE	V341I	23.363	0.045	Increasing
ALA	522	SER	A522S	41.819	0.088	Increasing
ALA	435	SER	A435S	15.197	0.148	Increasing
ASN	354	ASP	N354D	20.704	0.168	Increasing
ALA	520	SER	A520S	42.138	0.177	Increasing
ALA	348	THR	A367T	16.047	0.204	Increasing
VAL	367	PHE	V367F	23.608	0.235	Increasing
GLN	414	GLU	Q414E	15.173	0.252	Increasing
ILE	468	PHE	I468F	14.973	0.256	Increasing
From 9 March, 2020 to 30 November, 2020						
PHE	490	LEU	F490L	3.825	-0.791	Decreasing
LYS	417	ARG	K417R	2.862	-0.487	Decreasing
ASN	439	LYS	N439K^§^	7.234	-0.331	Decreasing
ASN	354	LYS	N354K	20.704	-0.294	Decreasing
PHE	490	SER	F490S	3.825	-0.239	Decreasing
SER	373	LEU	S373L	14.013	-0.203	Decreasing
VAL	382	LEU	V382L	30.232	-0.202	Decreasing
GLU	484	LYS	E484K	4.184	-0.154	Decreasing
THR	385	ILE	T385I	28.446	-0.128	Decreasing
ARG	408	LYS	R408K	10.586	-0.127	Decreasing
SER	494	PRO	S494P	6.031	-0.095	Decreasing
VAL	483	ALA	V483A	9.719	-0.053	Decreasing
THR	478	LYS	T478K	7.571	-0.028	Decreasing
ALA	475	VAL	A475V^§^	2.813	-0.001	Decreasing
PRO	384	SER	P384S	26.132	0.01	Increasing
SER	469	PRO	S469P	12.248	0.02	Increasing
SER	459	TYR	S459Y	11.205	0.02	Increasing
ARG	346	LYS	R346K	16.314	0.022	Increasing
ASN	354	SER	N354S	20.704	0.033	Increasing
PRO	521	ARG	P521R	42.427	0.036	Increasing
SER	514	PHE	S514F	26.529	0.041	Increasing
ALA	522	SER	A522S^§^	41.819	0.088	Increasing
SER	477	ASN	S477N	4.97	0.227	Increasing
VAL	367	PHE	V367F^§^	23.608	0.235	Increasing
ALA	522	GLU	A522E	41.819	0.409	Increasing
ASN	501	THR	N501T	3.115	0.87	Increasing

^§^These four mutations were also predicted in early stage

Then, we monitored the mutations predicted. Countries with more than 100 strains of relative mutations before 30 May, 2021 were included. Of all 60 types of mutants, the number of E484K was the largest with 86 585 sequences, followed by S477N with 55 442 sequences (Table 2). Up to 30 May, 2021, E484K strains in Brazil and S477N in Australia accounted for more than 50%, while S477N strains accounted for more than 10% in Switzerland, France, and Luxembourg.

**Table 2:** Quantitative monitoring of SARS-CoV-2 strains with specific mutations

Mutant strains	Collected locations	Onset date^†^	Numbers before onset (%^§^)	Numbers till deadline^#^ (%^§^)	P*
(total numbers)					
E484K	USA	2020/8/10	15/60 253 (0.02)	37 830/519 493 (7.28)	< 0.001
(86 585)	Brazil	2020/10/14	15/3104 (0.48)	8001/12 725 (62.88)	< 0.001
	Japan	2020/12/8	16/17 633 (0.09)	5849/46 122 (12.68)	< 0.001
	Germany	2020/12/28	15/7313 (0.21)	3679/119 793 (3.07)	1.67E−73
	France	2021/1/6	19/5299 (0.36)	3114/37 205 (8.37)	2.72E−155
	South Africa	2020/10/10	15/2451 (0.61)	2675/6329 (42.27)	< 0.001
	Sweden	2021/1/18	15/3656 (0.41)	2351/54 991 (4.28)	6.95E−47
	Belgium	2021/1/10	16/3952 (0.4)	2187/22 666 (9.65)	8.51E−133
	United Kingdom	2020/12/12	16/145 591 (0.01)	2071/421 559 (0.49)	1.81E−237
	Canada	2020/12/14	20/16 160 (0.12)	1644/27 996 (5.87)	4.20E−296
S477N**	USA	2020/8/21	15/63 825 (0.02)	12 772/519 495 (2.46)	< 0.001
(55 442)	Australia	2020/5/28	17/3447 (0.49)	10 090/17 811 (56.65)	< 0.001
	Denmark	2020/8/17	33/3015 (1.09)	6194/101 999 (6.07)	6.24E−43
	Switzerland	2020/8/24	16/2422 (0.66)	5701/40 653 (14.02)	1.16E−126
	France	2020/7/29	19/2537 (0.75)	3853/37 205 (10.36)	1.64E−87
	United Kingdom	2020/4/12	15/20 890 (0.07)	3318/421 559 (0.79)	2.10E−49
	Germany	2020/9/10	15/3474 (0.43)	3191/119 797 (2.66)	3.40E−23
	Luxembourg	2020/10/7	15/423 (3.55)	1445/8248 (17.52)	4.51E−18
	Sweden	2020/8/11	16/876 (1.83)	1232/54 991 (2.24)	0.488828411
	Belgium	2020/9/15	22/1862 (1.18)	1229/22 666 (5.42)	< 0.001
T478K	USA	2020/11/23	16/43 020 (0.04)	13 072/519 493 (2.52)	< 0.001
(31 516)	United Kingdom	2020/12/31	15/172 126 (0.01)	10 085/421 559 (2.39)	< 0.001
	Mexico	2020/11/30	17/2845 (0.6)	4876/14 372 (33.93)	< 0.001
	India	2021/3/1	16/11 429 (0.14)	1719/22 009 (7.81)	7.92E−292
	Germany	2021/2/4	16/16 175 (0.1)	566/119 793 (0.47)	1.56E−15
	Canada	2021/1/19	16/19 501 (0.08)	374/27 996 (1.34)	4.64E−65
	Denmark	2021/4/5	15/79 383 (0.02)	258/101 999 (0.25)	2.80E−46
	Switzerland	2020/12/25	17/12 385 (0.14)	244/40 653 (0.6)	6.06E−13
	Sweden	2021/1/25	17/4328 (0.39)	182/54 991 (0.33)	0.493286089
	Japan	2021/4/16	18/40 879 (0.04)	154/46 122 (0.33)	9.30E−25
N439K	Denmark	2020/8/24	21/3277 (0.64)	5599/101 999 (5.49)	1.46E−51
(27 987)	Slovenia	2020/8/15	16/350 (4.57)	4555/11 747 (38.78)	4.87E−50
	United Kingdom	2020/3/24	16/8202 (0.2)	4459/421 559 (1.06)	3.11E−20
	Germany	2020/8/25	15/3338 (0.45)	3217/119 793 (2.69)	3.83E−22
	Sweden	2020/11/23	16/1325 (1.21)	1459/54 991 (2.65)	0.000464083
	Switzerland	2020/8/21	15/2355 (0.64)	1334/40 653 (3.28)	2.11E−17
	Austria	2020/11/2	18/1085 (1.66)	1302/15 060 (8.65)	1.29E−21
	Italy	2020/10/14	15/2291 (0.65)	972/28 589 (3.4)	1.97E−17
	Netherlands	2020/10/15	18/3257 (0.55)	468/33 758 (1.39)	1.63E−05
	Indonesia	2020/12/30	19/623 (3.05)	467/1773 (26.34)	2.40E−45
S494P	USA	2020/7/18	15/52 902 (0.03)	6420/519 493 (1.24)	8.00E−243
(8638)	United Kingdom	2020/8/14	15/49 206 (0.03)	937/421 559 (0.22)	1.60E−27
	Spain	2020/12/20	15/9540 (0.16)	376/29 902 (1.26)	1.90E−28
	Germany	2021/2/5	18/16 737 (0.11)	150/119 793 (0.13)	0.637633683
A520S**	USA	2020/6/9	21/35 378 (0.06)	2867/519 493 (0.55)	8.67E−55
(4113)	United Kingdom	2020/6/24	16/43 220 (0.04)	238/421 559 (0.06)	0.105089403
	Denmark	2020/8/31	91/3996 (2.28)	199/101 999 (0.2)	7.18E−57
F490S	Denmark	2021/3/29	25/76 052 (0.03)	650/101 999 (0.64)	5.25E−122
(3954)	USA	2020/12/7	15/109 237 (0.01)	632/519 493 (0.12)	2.62E−34
	Germany	2021/2/22	17/27 057 (0.06)	601/119 793 (0.5)	2.88E−33
	United Kingdom	2020/12/15	15/150 582 (0.01)	418/421 559 (0.1)	3.21E−37
	Israel	2021/1/7	18/2706 (0.67)	378/11 835 (3.19)	6.52E−17
	Chile	2021/3/7	15/1454 (1.03)	273/2193 (12.45)	6.14E−45
	Poland	2021/3/3	15/2917 (0.51)	267/12 832 (2.08)	1.04E−10
	Argentina	2021/2/26	16/2115 (0.76)	136/3148 (4.32)	2.71E−16
	Netherlands	2021/3/31	18/23 847 (0.08)	116/33 758 (0.34)	2.98E−12
N501T**	USA	2020/8/28	15/65 569 (0.02)	2339/519 494 (0.45)	2.85E−97
(3288)	France	2021/1/9	23/5626 (0.41)	200/37 177 (0.54)	0.233423368
	Australia	2020/12/16	15/16 685 (0.09)	170/17 811 (0.95)	1.47E−32
A522S**	Germany	2020/12/10	15/6881 (0.22)	846/119 793 (0.71)	6.05E−08
(3275)	USA	2020/8/26	16/65 058 (0.02)	654/519 493 (0.13)	2.63E−17
	France	2021/1/27	23/7974 (0.29)	344/37 205 (0.92)	2.22E−10
	Denmark	2020/11/23	35/12 200 (0.29)	231/101 999 (0.23)	0.19572294
	United Kingdom	2020/8/31	20/54 038 (0.04)	231/421 559 (0.05)	0.091287615
	Russia	2020/11/9	17/1896 (0.9)	182/3962 (4.59)	1.05E−15
	Italy	2021/3/13	16/15 095 (0.11)	147/28 589 (0.51)	3.09E−13
	Austria	2021/1/18	19/2638 (0.72)	122/15 060 (0.81)	0.722036891
V367F**	United Kingdom	2020/4/17	15/24 276 (0.06)	391/421 559 (0.09)	0.126000419
(1886)	USA	2020/6/1	15/33 267 (0.05)	358/519 493 (0.07)	0.125838334
	Canada	2021/1/2	17/17 660 (0.1)	275/27 996 (0.98)	5.81E−39
	Uganda	2020/8/15	22/95 (23.16)	220/428 (51.4)	4.08E−07
A522V**	USA	2020/7/13	15/51 506 (0.03)	365/519 493 (0.07)	0.000214014
(1012)	United Kingdom	2020/4/7	15/17 505 (0.09)	197/421 559 (0.05)	0.03237495

*Fisher's exact test was used to compare proportion of mutant strains among all uploaded isolates which were collected between before onset date and till deadline

**These mutations are with increasing predicted affinity to receptor

^†^The onset date listed in the table was the time when more than 15 mutants were uploaded locally

^§^Proportion referred to the ratio of numbers of specific mutations among all uploaded sequences

^#^Deadline referred to 30 May, 2021

### Effects of SARS-CoV-2 spike mutations on viral infectivity and the reactivity to the neutralizing antibody

**Fig. 5:**
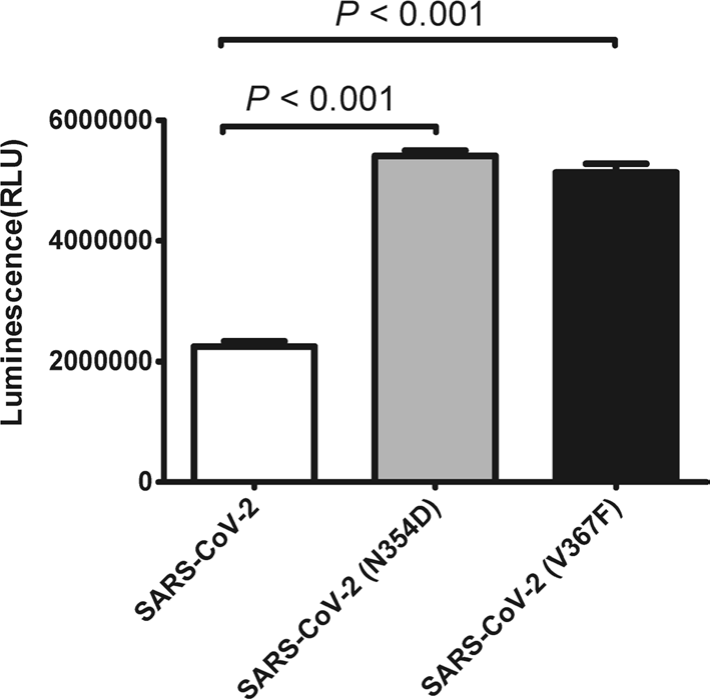
The effect of SARS-CoV-2 spike protein mutations on viral infectivity. HEK293T-ACE2 cells were infected with the recombinant lentiviruses’ pseudoviruses (0.37 µl, 1 × 10^7^ TU/ml) with the indicated SARS-CoV-2 S protein (wild-type, N354D, or V367F). The y-axis shows the relative luminescence units (RLU) detected at 48 h post-pseudovirus inoculation

**Fig. 6:**
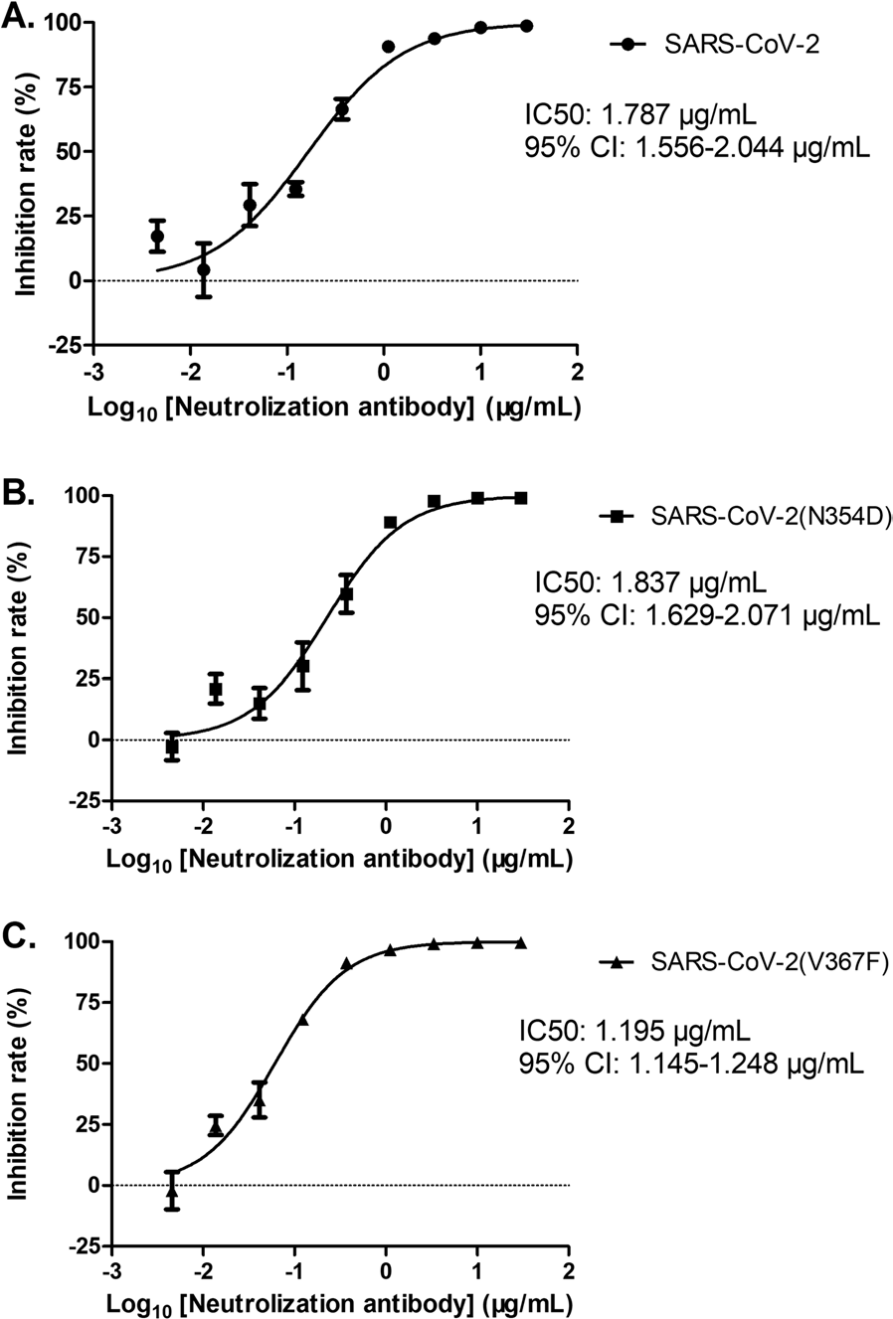
The effect of SARS-CoV-2 spike protein mutations on the reactivity to the neutralizing antibody. Pseudoviruses with the indicated SARS-CoV-2 S proteins (wild-type, N354D, or V367F) were incubated (1 h, room temperature) with different concentrations of neutralization antibody, before being inoculated into HEK293T-ACE2 cells. Efficiency of the transduction was quantified by testing the virus-encoded luciferase activity at 48 h post-transduction. For normalization, inhibition of pseudovirus transduction in HEK293T-ACE2 cells without neutralization antibody was set as 0%. The data are presented as the mean percentages of inhibition, and error bars indicate standard deviation. *RLU* relative luminescent unit

We infected HEK293T cells with SARS-CoV-2 pseudoviruses (wild-type, V367F mutant, and N354D mutant), and then tested the infectivity and immune reactivity. The V367F mutant (5.132 × 10^6^ RLU) and the N354D mutant (5.408 × 10^6^ RLU) were more highly infectious than the wild-type counterpart (2.243 × 10^6^ RLU) (Fig. 5). The immune reactivity was evaluated using SARS-CoV-2 S neutralizing antibody. The N354D mutant and wild-type counterpart showed a similar sensitivity to neutralizing antibody, while the V367F mutant was more sensitive to neutralizing antibody than wild-type counterpart (*P* < 0.001) (Fig. 6).

## Discussion

In this study, we analyzed the origination and evolution of SARS-CoV-2 using public databases and experiments in vitro. In the bioinformatic parts, we offered a pipeline to analyze the dynamics of SARS-CoV-2 evolution globally according to viruses’ genome, collecting details including geographic and temporal information. The results were also combined with the recombination analysis, the affinity prediction, and the quantitative monitoring of sequences to depict the nature of SARS-CoV-2 evolution. This pipeline of “evolutionary dynamics” helps identify the origination and transmission pattern of SARS-CoV-2.

Our recombination analysis of SARS-CoV-2 among CoVs from animals indicated that the nucleotide variations of CoVs were equally distributed in their genomes, without insertion or recombination of large fragment(s). The two possible recombination events are less likely to be real because of geographic isolation (Fig. 1). To the best of our knowledge, no evidence proved artificial modification on SARS-CoV-2. Our data support the result of a previous sequence analysis that SARS-CoV-2 should come from natural origin and evolution [24], which is also supported by the WHO report: the spillover of SARS-CoV-2 to human was likely through direct zoonotic transmission or intermediate host but was extremely unlikely due to a laboratory incident (https://www.who.int/publications/i/item/who-convened-global-study-of-origins-of-sars-cov-2-china-part). Thus, SARS-CoV-2 might come from natural hosts, rather than a man-made CoV.

This evolutionary dynamics provides evidence to determine the origins and transmission of SARS-CoV-2. SARS-CoV-2 strains clustered together are more likely to transmit each other. At the early stage, the strains identified in China, Japan, the USA, Singapore, Australia, Malaysia, and Italy clustered together as Cluster C (Fig. 3), indicating that strains could transmit each other. Strains in Cluster B which was distinct from Cluster C were identified in the USA, Canada, and Australia, indicating this clade is unlikely to be transmitted by the strains identified in China. During the whole process of this period, virus collected in China mainly gathered in one clade and had no strong links with other clusters. As the location with large number of isolates, USA had various kinds of mutant strains which formed at least 4 clades at the same time. According to the transmission network of early stage, no single and obvious source nodes were observed. These data imply that SARS-CoV-2 in China might be introduced from other countries.

In the USA, the first COVID-19 case was diagnosed on January 19, 2020 [6]. However, a recent study indicated that of 7389 routine blood donations in nine states of the USA from December 13, 2019 to January 17, 2020, 1.3% were seropositive for neutralizing antibody against SARS-CoV-2 [25], indicating that SARS-CoV-2 might transmit in the USA prior to January 19, 2020. Retrospective detection of SARS-CoV-2 genome in respiratory samples of symptomatic patients without relevant travel history indicated that patient tested positive for SARS-CoV-2 in the USA was identified on January 13, 2020 [26]. In Europe, the blood samples collected on November 4, 2019 in France and September to November 25, 2019 in Italy were positive for the antibody against SARS-CoV-2 [27–29], prior to the outbreak of COVID-19 in China [4]. SARS-CoV-2 genomic RNA can be detected in sewage systems of different countries during COVID-19 outbreak [30–32]. Importantly, SARS-CoV-2 genomic RNA was detected in waste water samples collected on 18 December, 2019 in Italy [33]. Cold-chain delivery of imported fresh seafood was the major way of introducing SARS-CoV-2 into cities including Beijing, Qingdao, Tianjin, and Dalian after May 2020 when the outbreak was well controlled in China [34–36]. COVID-19 outbreak occurred during the Spring Festival season. People routinely buy imported seafood to celebrate this holiday. Although nucleic acid test of SARS-CoV-2 was positive in environmental samples from stalls related to patients, SARS-CoV-2 was tested negative in wild animals in the Huanan seafood market. Furthermore, a total of 38 515 livestock and poultry samples and 41 696 wild animal samples from 31 provinces in China during 2018–2020 were tested negative for the antibody against SARS-CoV-2 or tested negative for SARS-CoV-2 nucleic acids (https://www.who.int/publications/i/item/who-convened-global-study-of-origins-of-sars-cov-2-china-part). Our data, together with the reported evidences, imply that SARS-CoV-2 might originate in several geographic areas including Europe, America, and Asia simultaneously under certain evolutionary pressure. China might not be the original location where the spillover of SARS-CoV-2 from wildlife to humans occurs. The ancestors of SARS-CoV-2 might circulate among natural reservoirs and keep evolving in given ecological environments. The spillover to humans might be a specific stage during evolutionary course, just like SARS-CoV-1 that has disappeared for > 17 years.

After the spillover, SARS-CoV-2 strains in different countries had their own directions of evolution, rendering increasingly obvious trends of location-based gathering. The colors of different clusters became “purer” during the global pandemic, with fewer nodes of mixed colors (Fig. 4). Appropriate control strategies from governments help prevent the pandemic [37, 38]. Travel restrictions were implemented across the world [39]. After the international travel restrictions, the strains clustered locally and the risks of introducing mutant strains decreased in given countries. Since May 2020, India and South Africa reported a large number of clustered strains. Viruses identified in the two countries played key roles in forming the core of clusters in the transmission network. The mutant strains in both countries showed possible higher infectivity and antigenicity than SARS-CoV-2 strains at the early stage [40, 41]. Mutant strains including B.1.617 were epidemic in India, according to the reports of the WHO. Meanwhile, mutant strains identified in South Africa include B.1.351, a strain reported in late 2020 [41]. The time points of the mutant epidemic were consistent with the improved clustering of SARS-CoV-2 in both countries.

The affinity of mutated RBD region to ACE2 was predicted and mutated sequences throughout the pandemic were quantitated in this study. The mutant in RBD may lead to altered ACE2-binding ability and altered antigenicity [42]. We then monitored those mutations until 30 May, 2021. We found that 60 amino acid mutations of the S protein might alter SARS-CoV-2 transmission (Table 1). Of those, E484K was the most frequent one (*n* = 86 585). E484K was associated with a decreased affinity (Table 2), which is consistent with a previous report [43]. However, E484K lead to immune evasion from both natural and vaccine-induced sera [44, 45]. S477N, a mutation mainly identified in the USA, Australia, and some European countries, also had a large number of uploaded sequences. S477N enhance the binding affinity [45]. It was reported that COVID-19 influenced the host immunity [46]. Such process might be altered by mutant SARS-CoV-2, inducing more severe cases or wider epidemic. Thus, rapid identification of emerging mutants with immune evasion including E484K and those with increased binding affinity such as S477N is important in tracing SARS-CoV-2 evolution.

I468F, Q414E, V367F, A367T, A520S, N354D, and A435S were identified to be the early mutations with the affinity change of ΔΔG_wild-mutation_ > 0.1 kcal/mol (Table 1). Of those, the sequences with I468F, Q414E, A367T, or A435S were not chosen due to only < 10 strains uploaded in 2020. A520S was reported to be associated with low antigenicity [47]. V367F was present at the early stage and thereafter. Thus, V367F and N354D were selected for the in vitro experiments. It was demonstrated that V367F and N354D mutants showed higher infectivity than wild-type counterpart (Fig. 5). For the first time, we demonstrated that the V367F mutant exhibits more sensitivity to the neutralizing antibody than wild-type counterpart (*P* < 0.001), possibly because this mutation increases the antigenicity [47]. Although V367F increases its binding affinity to ACE2, it increases the reactivity to neutralizing antibody. Thus, the proportion of this mutant did not increase significantly during the pandemic in Western world (Table 2). SARS-CoV-2 particles contain 24 ± 9 S trimers [48]. It remains to be clarified if SARS-CoV-2 mutations might influence the antigenicity via affecting the conformation and number of trimers of SARS-CoV-2 particles. The neutralizing antibody applied in this study is a kind of monoclonal antibody targeting to the S1 protein, which has a higher reactivity to the V367F-related antigenic determinant. In most cases, however, SARS-CoV-2 mutations facilitate escape from antibody neutralization [49]. The combined application of two or more neutralizing antibodies to SARS-CoV-2 S protein can prevent the mutated viruses [50, 51]. N354D mutation increased the infectivity of SARS-CoV-2, but did not alter antibody neutralization. These data indicate that the association of SARS-CoV-2 mutations with antibody neutralization are complicated and need extensively epidemiological studies.

Our study has limitations. First, the effect of combined SARS-CoV-2 mutations was not evaluated due to lack of suitable methods. SARS-CoV-2 mutants that acquire several immune escape mutations may be highly infectious. Second, the effects of the SARS-CoV-2 mutations on the conformation and number of trimers of SARS-CoV-2 are not evaluated in this study. Third, SARS-CoV-2 sequences were often identified and uploaded in countries with a higher level of academic activity, thus introducing a selection bias. Finally, the numbers of uploaded strains were not consistent with the actual case number.

## Conclusions

Conclusively, the present study indicates that SARS-CoV-2 strains might have originated in several countries simultaneously under certain evolutionary pressure. Continent- and country-specific clustering of SARS-CoV-2 strains might be caused by travel restrictions. SARS-CoV-2 evolution affects the transmission via altering the affinity to ACE2, immune escape, and possibly viral replication. The method of evolutionary dynamics in this study can be applied to trace the transmission and predict key SARS-CoV-2 mutations worldwide in the future.

## Supplementary Information

**Additional file 1: Table S1.** Sequences included for detecting genetic recombination relevant to SARS-CoV-2.

**Additional file 2: Figure S1.** DNA sequencing to verify V367F and N354D mutations in the pseudovirus genome.

**Additional file 3: Figure S2.** Network graphic of SARS-CoV-2 isolates worldwide during 9 and 31 March 2020. Isolates were aligned by the Force Atlas model in Gephi. In the network, each node represented an isolate of SARS-CoV-2. Each color represented a country. Lines inherit colors from their origin clades. Distances between clades represented evolutionary distance.

**Additional file 4: Figure S3.** Network graphic of SARS-CoV-2 isolates worldwide during 1 and 30 April 2020. Isolates were aligned by the Force Atlas model in Gephi. In the network, each node represented an isolate of SARS-CoV-2. Each color represented a country. Lines inherit colors from their origin clades. Distances between clades represented evolutionary distance.

**Additional file 5: Figure S4.** Network graphic of SARS-CoV-2 isolates worldwide during 1 May and 30 June 2020. Isolates were aligned by the Force Atlas model in Gephi. In the network, each node represented an isolate of SARS-CoV-2. Each color represented a country. Lines inherit colors from their origin clades. Distances between clades represented evolutionary distance.

**Additional file 6: Figure S5.** Network graphic of SARS-CoV-2 isolates worldwide during 1 July and 31 August 2020. Isolates were aligned by the Force Atlas model in Gephi. In the network, each node represented an isolate of SARS-CoV-2. Each color represented a country. Lines inherit colors from their origin clades. Distances between clades represented evolutionary distance.

## Data Availability

All data generated or analysed during this study were kept by Department of Epidemiology, Second Military Medical University, Shanghai, China. Please contact author for data requests.

## Abbreviations

CoVs: Coronaviruses
SARS-CoV: Severe acute respiratory syndrome CoV
MERS-CoV: Middle East respiratory syndrome CoV
SARS-CoV-2: Severe acute respiratory syndrome-related coronavirus-2
ACE2: Angiotensin converting enzyme II
WHO: World Health Organization
DMEM: Dulbecco's modified Eagle's medium
FBS: Fetal bovine serum
SPSS: Statistical Package for Social Sciences
*SD*s: Standard deviations
S: Spike
RBD: Receptor-binding domain
RDP: Recombination Detection Program
ORFs: Open reading frames
VSV: Vesicular stomatitis virus
TU/ml: Transduction units per milliliter
RLUs: Relative luminescent units
