# Supplementary material for: Characterization of SARS-CoV-2 worldwide transmission based on evolutionary dynamics and specific viral mutations in the spike protein
Source: Infect Dis Poverty. 2021 Aug 21;10:112. doi: 10.1186/s40249-021-00895-4 (PMC8379592; doi:10.1186/s40249-021-00895-4)
Supplement: Supplementary file 1 — Additional file 1: Table S1. Sequences included for detecting genetic recombination relevant to SARS-CoV-2. [file 40249_2021_895_MOESM1_ESM.docx]

**Table S1. Sequences included for detecting genetic recombination relevant to SARS-CoV-2.**

| Accession Number | Sequence name | Database | Type of CoV | Collection date | Location | Host | |
| --- | --- | --- | --- | --- | --- | --- | --- |
|  |  |  |  |  |  | Species | Common name |
| MN996532 | Bat coronavirus RaTG13 | GISAID / Genbank | Unclassified | 2013 | Yunnan/China | Rhinolophus affinis | Bat |
| EF065509 | Bat coronavirus HKU5-1 | Genbank | Betacoronavirus | 2006 | Guangdong/China | Rhinolophus affinis | Bat |
| EF065510 | Bat coronavirus HKU5-2 | Genbank | Betacoronavirus | 2006 | Guangdong/China | Rhinolophus affinis | Bat |
| EF065511 | Bat coronavirus HKU5-3 | Genbank | Betacoronavirus | 2006 | Guangdong/China | Rhinolophus affinis | Bat |
| EF065512 | Bat coronavirus HKU5-5 | Genbank | Betacoronavirus | 2006 | Guangdong/China | Rhinolophus affinis | Bat |
| GU190215 | Bat coronavirus BM48-31/BGR/2008 | Genbank | Unclassified | 2008 | Bulgaria | Rhinolophus affinis | Bat |
| KC881005 | Bat SARS-like coronavirus RsSHC014 | Genbank | Betacoronavirus | 2011 | China | Rhinolophus affinis | Bat |
| KC881006 | Bat SARS-like coronavirus Rs3367 | Genbank | Betacoronavirus | 2012 | China | Rhinolophus affinis | Bat |
| KX574227 | Bat coronavirus isolate PREDICT/PDF-2180 | Genbank | Unclassified | 2013 | Uganda | Pipistrellus | Bat |
| KY417143 | Bat SARS-like coronavirus isolate Rs4081 | Genbank | Betacoronavirus | 2012 | China | Rhinolophus affinis | Bat |
| KY417146 | Bat SARS-like coronavirus isolate Rs4231 | Genbank | Betacoronavirus | 2013 | China | Rhinolophus affinis | Bat |
| KY417148 | Bat SARS-like coronavirus isolate Rs4247 | Genbank | Betacoronavirus | 2013 | China | Rhinolophus affinis | Bat |
| KY938558 | Bat coronavirus strain 16BO133 | Genbank | Unclassified | 2016 | South Korea | Rhinolophus ferrumequinum | Horseshoe bat |
| MG772933 | Bat-SL-CoVZC45 | Genbank | Betacoronavirus | 2017 | China | Rhinolophus affinis | Bat |
| MG772934 | Bat-SL-CoVZXC21 | Genbank | Betacoronavirus | 2018 | China | Rhinolophus affinis | Bat |
| NC014470 | Bat coronavirus BM48-31/BGR/2008 | Genbank | Unclassified | 2008 | Bulgaria | Rhinolophus blasii | Bat |
| FJ376620 | Bulbul coronavirus HKU11-796 | Genbank | Deltacoronavirus | 2007 | Hongkong/China | Chinese bulbul | Chinese bulbul |
| NC11547 | Bulbul coronavirus HKU11-934 | Genbank | Deltacoronavirus | 2007 | Hongkong/China | Red-whiskered bulbul | Red-whiskered bulbul |
| JX860640 | Canine respiratory coronavirus strain K37 | Genbank | Betacoronavirus | 2008 | South Korea | Canine | Dog |
| KX432213 | Canine respiratory coronavirus strain BJ232 | Genbank | Betacoronavirus | 2014 | China | Canine | Dog |
| KM347965 | Ferret coronavirus isolate FRCoV-NL-2010 | Genbank | Alphacoronavirus | 2010 | Netherlands | Mustela putorius | Ferret |
| KX512809 | Ferret enteric coronavirus strain FECV1 | Genbank | Alphacoronavirus | 2015 | Michigan/USA | Mustela putorius | Ferret |
| KX512810 | Ferret systemic coronavirus strain FSCV6 | Genbank | Alphacoronavirus | 2015 | Michigan/USA | Mustela putorius | Ferret |
| LC119077 | FRCoV4370 | Genbank | Alphacoronavirus | 2013 | Japan | Mustela putorius | Ferret |
| LC215871 | Ferret063 | Genbank | Alphacoronavirus | 2016 | Japan | Mustela putorius | Ferret |
| MT084071 | Pangolin coronavirus isolate MP789 | Genbank | Betacoronavirus | 2019 | China | Manis javanica | Pangolin |
| MH002341 | Pipistrellus bat HKU5 isolate BY140562 | Genbank | Betacoronavirus | 2014 | China | Pipistrellus | Bat |
| AY278741 | SARS coronavirus Urbani | Genbank | Betacoronavirus | No records | No records | Homo sapiens | Human |
| AY283798 | SARS coronavirus Sin2774 | Genbank | Betacoronavirus | 2003 | Singapore | Homo sapiens | Human |
| AY291315 | SARS coronavirus Frankfurt 1 | Genbank | Betacoronavirus | 2003 | Frankfurt | Homo sapiens | Human |
| AY291451 | SARS coronavirus TW1 | Genbank | Betacoronavirus | 2003 | Taiwan/China | Homo sapiens | Human |
| AY515512 | SARS coronavirus HC/SZ/61/03 | Genbank | Betacoronavirus | 2003 | Shenzhen/China | Paguma larvata | Masked civet |
| AY545914 | SARS coronavirus isolate HC/SZ/79/03 | Genbank | Betacoronavirus | 2003 | Shenzhen/China | Paguma larvata | Masked civet |
| AY572038 | SARS coronavirus civet020 | Genbank | Betacoronavirus | 2004 | China | Paguma larvata | Masked civet |
| AY654624 | SARS coronavirus TJF | Genbank | Betacoronavirus | 2003 | Tianjin/China | Swine | Pig |
| AY864805 | SARS coronavirus BJ162 | Genbank | Betacoronavirus | 2004 | Beijing/China | Homo sapiens | Human |
| FJ882945 | SARS coronavirus MA15 isolate P3pp6 | Genbank | Betacoronavirus | 2008 | Tennessee/USA | VeroE6 cells, BALB/c mice |  |
| FJ882948 | SARS coronavirus MA15 isolate P3pp3 | Genbank | Betacoronavirus | 2008 | Tennessee/USA | VeroE6 cells, BALB/c mice |  |
| FJ882952 | SARS coronavirus MA15 isolate P3pp4 | Genbank | Betacoronavirus | 2008 | Tennessee/USA | VeroE6 cells, BALB/c mice |  |
| JX163923 | SARS coronavirus isolate Tor2/FP1-10912 | Genbank | Betacoronavirus | 2010 | USA | Ferrets vaccinated |  |
| JX163924 | SARS coronavirus isolate Tor2/FP1-10851 | Genbank | Betacoronavirus | 2010 | USA | Ferrets vaccinated |  |
| AY274119 | SARS-related coronavirus isolate Tor2 | Genbank | Betacoronavirus | 2003 | Toronto/Canada | Homo sapiens | Human |
| KY352407 | SARS-related coronavirus BtKY72 | Genbank | Betacoronavirus | 2007 | Kenya | Rhinolophus affinis | Bat |
| MN988668 | 2019-nCoV WHU01 | Genbank | Betacoronavirus | 2020 | Wuhan/China | Homo sapiens | Human |
| MT093571 | SARS-CoV-2/01/human/2020/SWE | Genbank | Betacoronavirus | 2020 | Sweden | Homo sapiens | Human |
| FJ376621 | Thrush coronavirus HKU12-600 | Genbank | Deltacoronavirus | 2007 | Hongkong/China | Grey-backed thrush | Grey-backed thrush |
| JQ065044 | White-eye coronavirus HKU16-6847 | Genbank | Deltacoronavirus | 2007 | Hongkong/China | White-eye | White-eye |
| NC045512 | Wuhan-Hu-1 | Genbank | Betacoronavirus | 2019 | Wuhan/China | Homo sapiens | Human |
| EPI_ISL_410721 | Pangolin/Guangdong/China | GISAID | Betacoronavirus | 2019 | Guangdong/China | Manis javanica | Pangolin |
| EPI_ISL_410539 | Pangolin/Guangxi/China | GISAID | Betacoronavirus | 2017 | Guangxi/China | Manis javanica | Pangolin |
| EPI_ISL_410540 | Pangolin/Guangxi/China | GISAID | Betacoronavirus | 2017 | Guangxi/China | Manis javanica | Pangolin |
