# Supplementary figures and images for: Characterization of SARS-CoV-2 worldwide transmission based on evolutionary dynamics and specific viral mutations in the spike protein
Source: Infect Dis Poverty. 2021 Aug 21;10:112. doi: 10.1186/s40249-021-00895-4 (PMC8379592; doi:10.1186/s40249-021-00895-4)

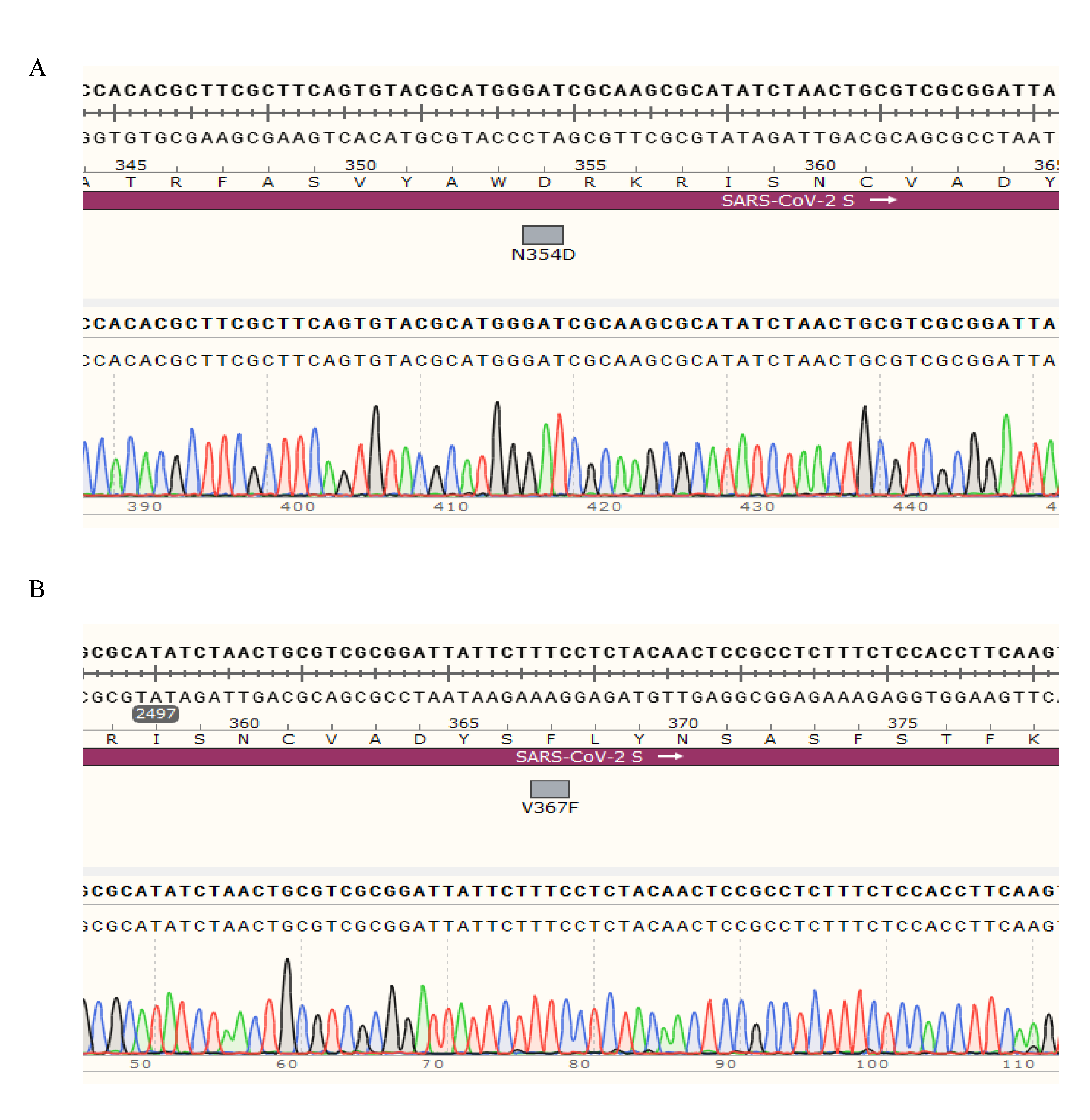

Supplement: Supplementary file 2 — Additional file 2: Figure S1. DNA sequencing to verify V367F and N354D mutations in the pseudovirus genome. [file 40249_2021_895_MOESM2_ESM.tif]

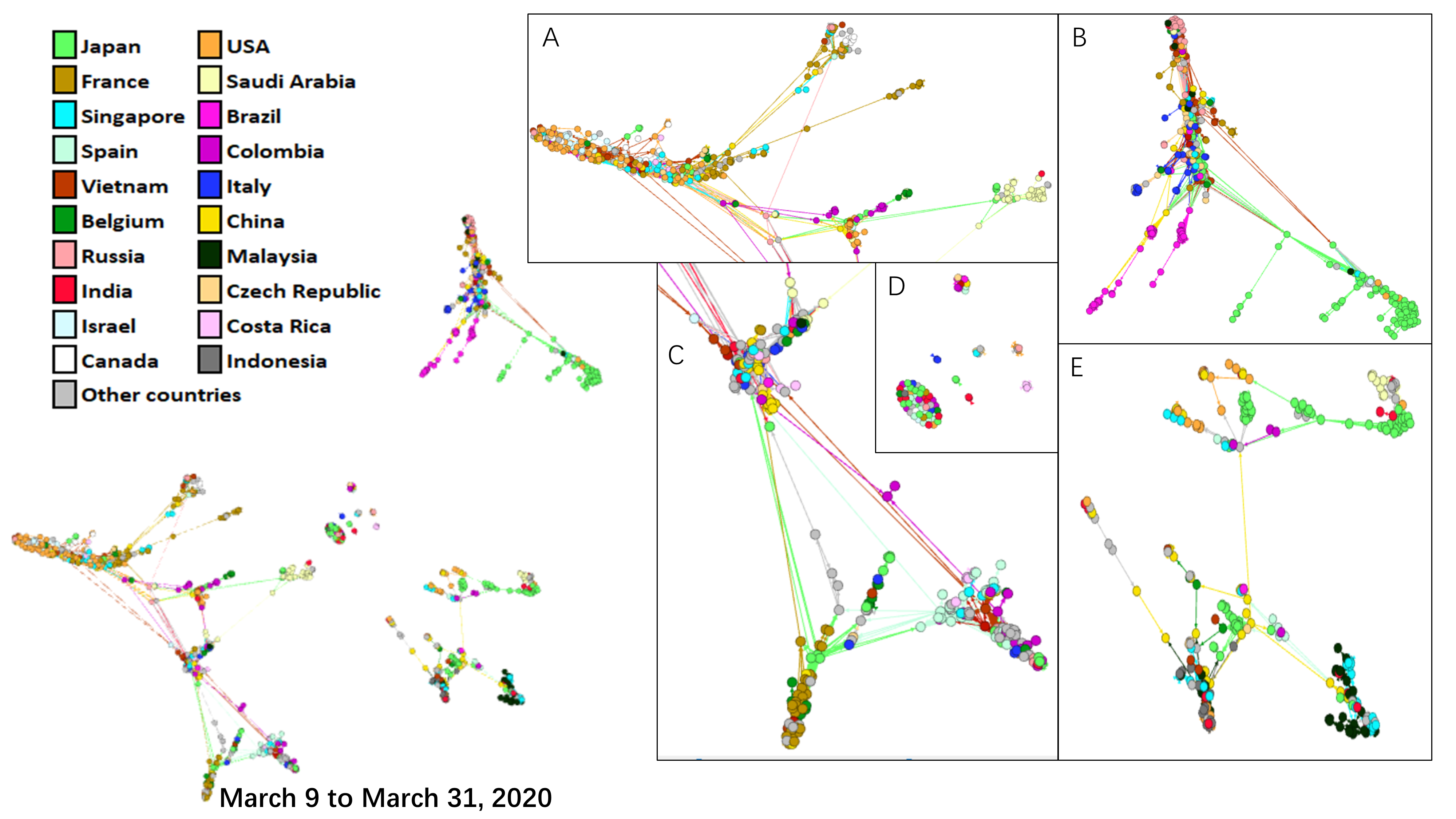

Supplement: Supplementary file 3 — Additional file 3: Figure S2. Network graphic of SARS-CoV-2 isolates worldwide during 9 and 31 March 2020. Isolates were aligned by the Force Atlas model in Gephi. In the network, each node represented an isolate of SARS-CoV-2. Each color represented a country. Lines inherit colors from their origin clades. Distances between clades represented evolutionary distance. [file 40249_2021_895_MOESM3_ESM.tif]

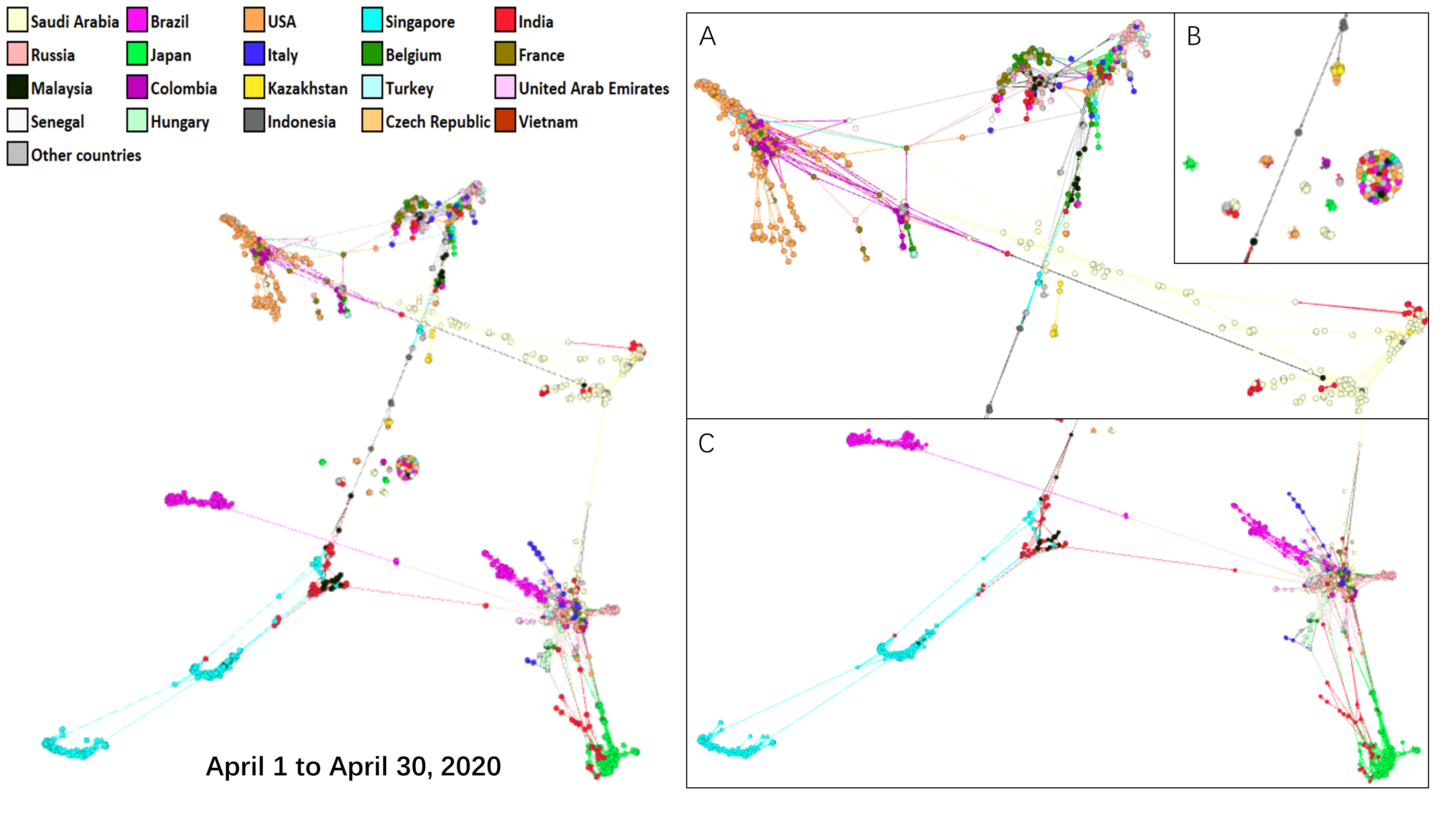

Supplement: Supplementary file 4 — Additional file 4: Figure S3. Network graphic of SARS-CoV-2 isolates worldwide during 1 and 30 April 2020. Isolates were aligned by the Force Atlas model in Gephi. In the network, each node represented an isolate of SARS-CoV-2. Each color represented a country. Lines inherit colors from their origin clades. Distances between clades represented evolutionary distance. [file 40249_2021_895_MOESM4_ESM.tif]

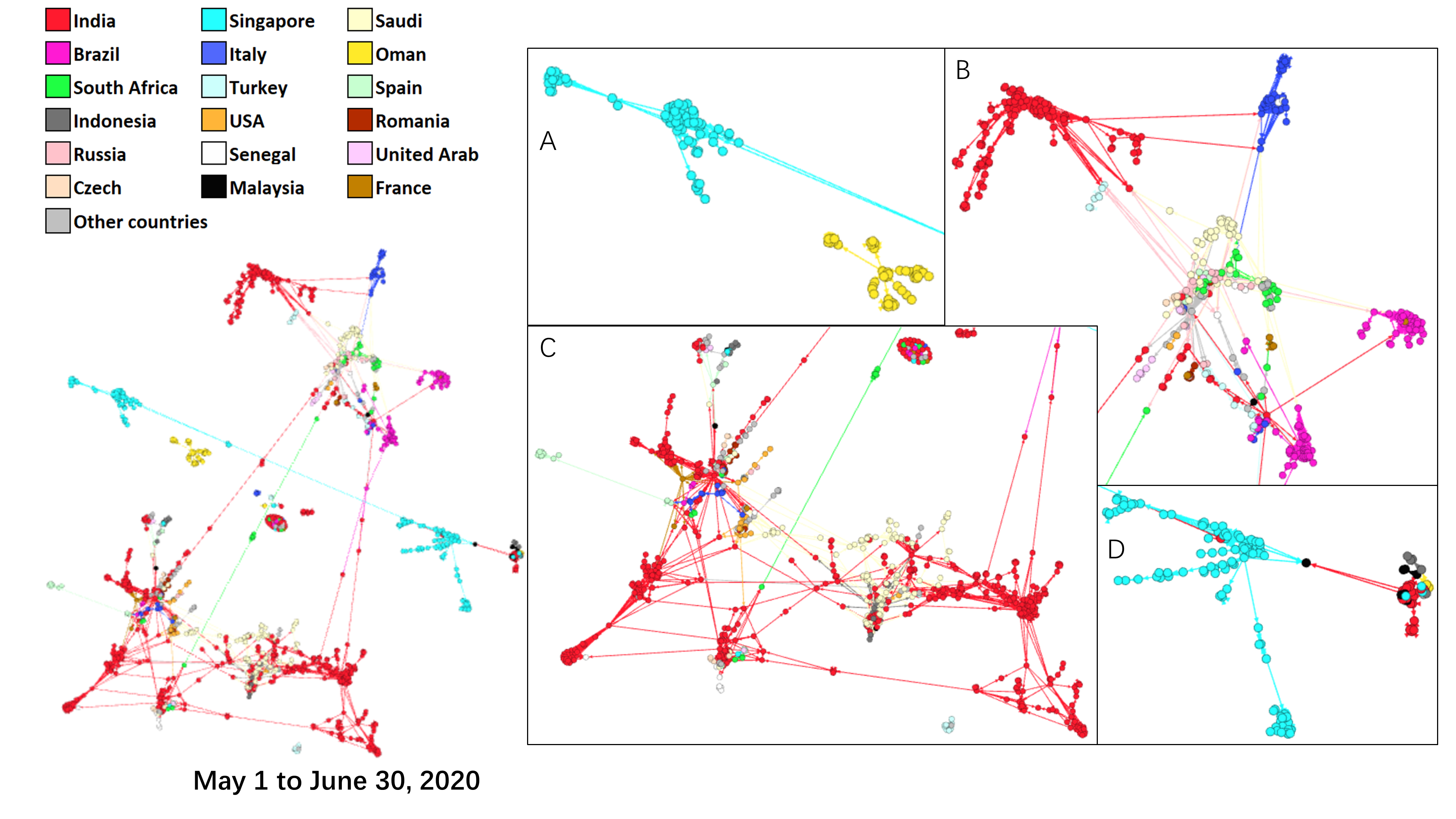

Supplement: Supplementary file 5 — Additional file 5: Figure S4. Network graphic of SARS-CoV-2 isolates worldwide during 1 May and 30 June 2020. Isolates were aligned by the Force Atlas model in Gephi. In the network, each node represented an isolate of SARS-CoV-2. Each color represented a country. Lines inherit colors from their origin clades. Distances between clades represented evolutionary distance. [file 40249_2021_895_MOESM5_ESM.tif]

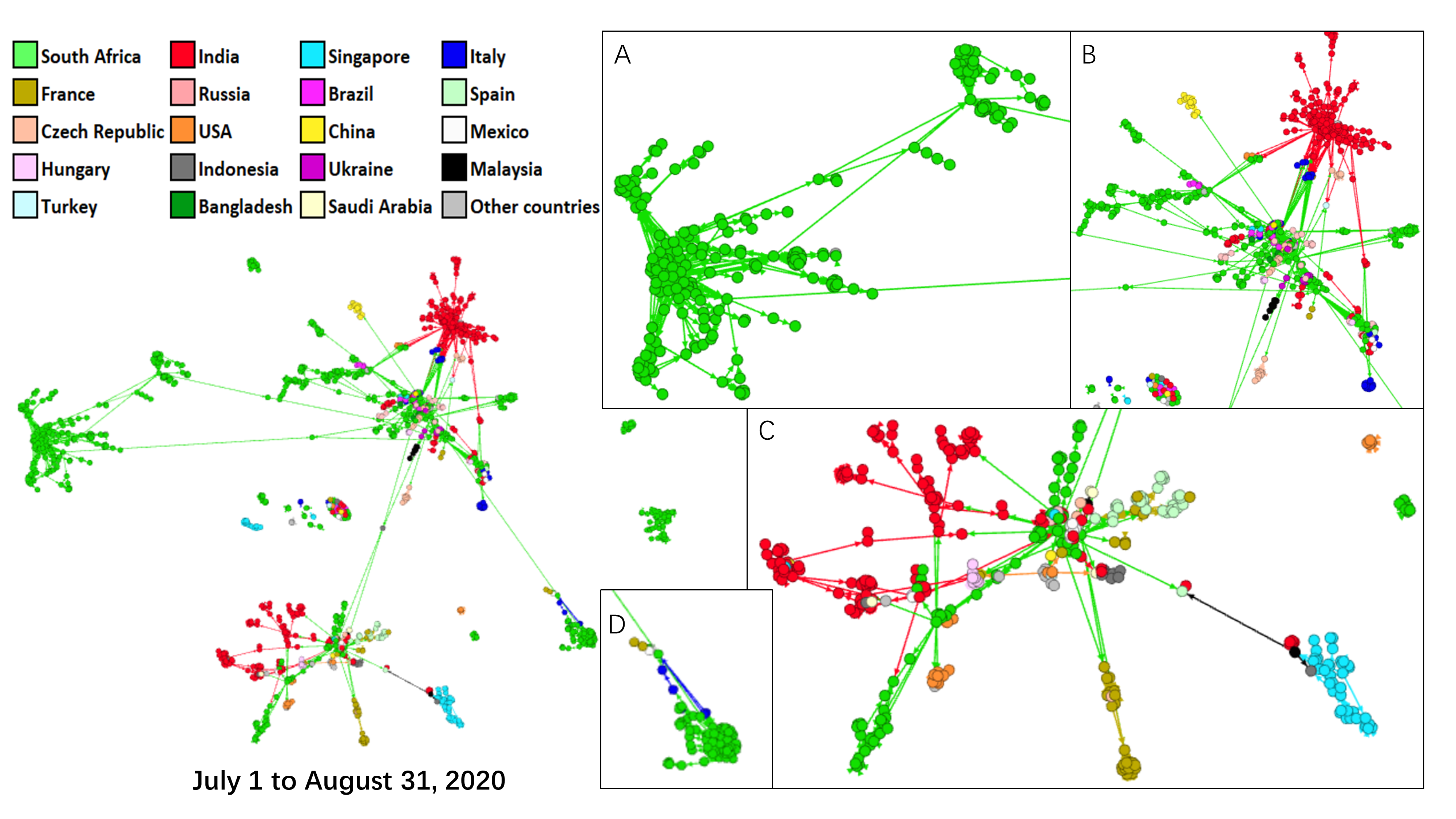

Supplement: Supplementary file 6 — Additional file 6: Figure S5. Network graphic of SARS-CoV-2 isolates worldwide during 1 July and 31 August 2020. Isolates were aligned by the Force Atlas model in Gephi. In the network, each node represented an isolate of SARS-CoV-2. Each color represented a country. Lines inherit colors from their origin clades. Distances between clades represented evolutionary distance. [file 40249_2021_895_MOESM6_ESM.tif]
